# Variable Abundance and Distribution of *Wolbachia* and *Cardinium* Endosymbionts in Plant-Parasitic Nematode Field Populations

**DOI:** 10.3389/fmicb.2019.00964

**Published:** 2019-05-07

**Authors:** Sulochana K. Wasala, Amanda M. V. Brown, Jiwon Kang, Dana K. Howe, Amy B. Peetz, Inga A. Zasada, Dee R. Denver

**Affiliations:** ^1^Department of Integrative Biology, Oregon State University, Corvallis, OR, United States; ^2^Department of Biological Sciences, Texas Tech University, Lubbock, TX, United States; ^3^USDA-ARS Horticultural Crops Research Laboratory, Corvallis, OR, United States

**Keywords:** bacterial endosymbionts, nematode, sex ratio distortion, *Wolbachia*, *Cardinium*, *Pratylenchus penetrans*

## Abstract

The bacterial endosymbiont *Wolbachia* interacts with different invertebrate hosts, engaging in diverse symbiotic relationships. *Wolbachia* is often a reproductive parasite in arthropods, but an obligate mutualist in filarial nematodes. *Wolbachia* was recently discovered in plant-parasitic nematodes, and, is thus far known in just two genera *Pratylenchus* and *Radopholus*, yet the symbiont’s function remains unknown. The occurrence of *Wolbachia* in these economically important plant pests offers an unexplored biocontrol strategy. However, development of *Wolbachia*-based biocontrol requires an improved understanding of symbiont-host functional interactions and the symbiont’s prevalence among nematode field populations. This study used a molecular-genetic approach to assess the prevalence of a *Wolbachia* lineage (wPpe) in 32 field populations of *Pratylenchus penetrans.* Populations were examined from eight different plant species in Washington, Oregon, and California. Nematodes were also screened for the endosymbiotic bacterium *Cardinium* (cPpe) that was recently shown to co-infect *P. penetrans*. Results identified wPpe in 9/32 and cPpe in 1/32 of *P. penetrans* field populations analyzed. No co-infection was observed in field populations. *Wolbachia* was detected in nematodes from 4/8 plant-hosts examined (raspberry, strawberry, clover, and lily), and in all three states surveyed. *Cardinium* was detected in nematodes from mint in Washington. In the wPpe-infected *P. penetrans* populations collected from raspberry, the prevalence of wPpe infection ranged from 11 to 58%. This pattern is unlike that in filarial nematodes where *Wolbachia* is an obligate mutualist and occurs in 100% of the host. Further analysis of wPpe-infected populations revealed female-skewed sex ratios (up to 96%), with the degree of skew positively correlating with wPpe prevalence. Uninfected nematode populations had approximately equal numbers of males and females. Comparisons of 54 wPpe 16S ribosomal RNA sequences revealed high similarity across the geographic isolates, with 45 of 54 isolates being identical at this locus. The complete absence of wPpe among some populations and low prevalence in others suggest that this endosymbiont is not an obligate mutualist of *P. penetrans*. The observed sex ratio bias in wPpe-infected nematode populations is similar to that observed in arthropods where *Wolbachia* acts as a reproductive manipulator, raising the question of a similar role in plant-parasitic nematodes.

## Introduction

Microbial symbiosis is increasingly recognized as an integral component of ecosystems with a major role in ecology and evolution. These symbiotic relationships range from mutualism (where both symbiotic partners benefit) to commensalism (where one symbiont benefits, while the other has no notable fitness effect) to parasitism (where one symbiont is harmed). Symbiotic relationships between microbes and multicellular hosts are often subdivided into ectosymbiosis such as gammaproteobacteria associated with the external surfaces of marine invertebrates (Goffredi, 2010), and endosymbiosis such as photosynthetic *Symbiodinium* within cnidarian cells and alphaproteobacteria within insect cells (Werren et al., 2008; Fransolet et al., 2012). Endosymbiosis involves intimate interactions between organisms, in which a microbial partner lives within its host cells. Many microbial symbioses likely originated as facultative relationships, which over time evolved into obligate relationships (Moya et al., 2008; Lamelas et al., 2011; Zug and Hammerstein, 2014). Further, eukaryotic organelles such as mitochondria and chloroplasts evolved from endosymbiotic events involving alphaproteobacterial and cyanobacterial ancestors, respectively. Microbial symbionts also serve as an exciting potential target for disease management and prevention. Despite the basic and applied importance of microbial symbiosis, ecological, and functional details remain to be understood in many systems.

*Wolbachia* is a common bacterial endosymbiont occurring in arthropods and nematodes. It was first detected in the mosquito *Culex pipiens* (Hertig, 1936) and since then has become established as a successful biocontrol agent for many pests and disease vectors (Brelsfoard and Dobson, 2009; Slatko et al., 2014). *Wolbachia* has varying interaction with invertebrate hosts, ranging from parasitic to mutualistic. In arthropods, *Wolbachia* typically acts as a reproductive parasite inducing a range of reproductive perturbations. *Wolbachia* induced reproductive abnormalities in arthropod hosts are often described in four categories: cytoplasmic incompatibility (CI), male-killing, genetic male feminization, and parthenogenesis-induction (Werren et al., 2008). *Wolbachia*-induced parthenogenesis is often observed in species with haplodiploid sex determination, in which males develop from unfertilized eggs and are haploid (Werren et al., 2008). Here, *Wolbachia* induces diploid development of unfertilized eggs so that infected females produce daughters instead of sons. Parthenogenesis-inducing *Wolbachia* phenotypes have been observed in *Trichogramma* wasps, mites, and thrips (Stouthamer et al., 1990; Arakaki et al., 2001; Weeks and Breeuwer, 2001). *Wolbachia*-induced feminization results in genetic males developing into functional females, as has been described in isopods, and insects in the orders Lepidoptera, and Hemiptera (Kageyama et al., 2002; Vandekerckhove et al., 2003; Negri et al., 2006; Kern et al., 2015). *Wolbachia* is also reported to selectively kill male hosts, mainly during embryogenesis (Werren et al., 2008), in the arthropod orders Coleoptera, Diptera, Lepidoptera, and in Pseudoscorpiones (Fialho and Stevens, 2000; Hurst et al., 2000; Jiggins et al., 2000; Dyson and Hurst, 2004; Zeh et al., 2005). *Wolbachia* primarily undergoes vertical transmission, through host eggs and not via sperm (Werren et al., 2008). Thus, the evolutionary advantages of these symbionts often involve improving transmission by increasing the proportion of females in the host population. Consequently, except for CI, the reproductive abnormalities induced by *Wolbachia* distort sex-ratio of the host population toward females (Cordaux et al., 2011).

CI is the most frequently observed *Wolbachia* phenotype in which sperm from *Wolbachia*-infected males are incompatible with eggs from females that do not harbor *Wolbachia* (or the same *Wolbachia* strain), such that resulting crosses result in no viable offspring. This phenomenon was first observed in the mosquito *C. pipiens* (Hertig, 1936), and has since been described in other insect orders, arachnids, and isopods (Bourtzis et al., 1996; Sicard et al., 2014; Xie et al., 2016). As a result of CI, *Wolbachia* in arthropods can cause a form of conditional sterility that can be used to suppress populations of medically and economically important insects (Slatko et al., 2014). For example, introduction of *Wolbachia* into medfly *Ceratitis capitate*, and disease-causing mosquito populations was shown to suppress those insect pest populations (Zabalou et al., 2004; Flores and O’Neill, 2018). By contrast, in animal and human-parasitic filarial nematodes, *Wolbachia* acts as an obligate mutualist and is required for normal host development, fertility, and survival. Treating filarial nematode-infected animals and humans with antibiotics that eliminate *Wolbachia* resulted in the prevention of nematode embryo development (Bandi et al., 1999), growth retardation/infertility (Hoerauf et al., 1999), and eventual death (Langworthy et al., 2000). Since treatments with antibiotics that eliminate mutualistic *Wolbachia* in filarial nematodes have adverse effects on the symbiont host, this approach has been used for the treatment of human filariasis (Taylor et al., 2005; Turner et al., 2017).

Previously, there was only one reported case of *Wolbachia* infecting a plant-parasitic nematode genus: *Radopholus* (burrowing nematode) (Haegeman et al., 2009). We recently discovered *Wolbachia* in another plant-parasitic nematode, *Pratylenchus penetrans* (root-lesion nematode), through genome skimming and fluorescence *in situ* hybridization (FISH) analyses (Brown et al., 2016; Denver et al., 2016). This *Wolbachia* strain was designated wPpe. *P. penetrans* is an amphimictic nematode species, with males and females reported as occurring in approximately equal numbers (Roman and Triantaphyllou, 1969), although there was one previously reported case of *P. penetrans* populations being female-biased (Tarte and Mai, 1976). The *P. penetrans* population in which wPpe was initially discovered, also carried another endosymbiotic bacterium Cardinium (cPpe) (Brown et al., 2016; Denver et al., 2016). *Cardinium* is often found with *Wolbachia* in arthropods but has not been reported to occur in filarial nematodes. However, *Cardinium* has been reported in two other plant-parasitic nematode genera; *Heterodera* (soybean-cyst nematode) (Shepherd et al., 1973; Endo, 1979; Noel and Atibalentja, 2006; Brown, 2018) and *Globodera* (potato-cyst nematode) (Walsh et al., 1983a,b; Brown, 2018).

Bacterial symbioses in plant-parasitic nematodes have generally received limited research attention, except for the bacterial genus *Pasteuria* (Atibalentja and Noel, 2008). However, plant-parasitic nematodes are responsible for more than US $100 billion in annual agriculture loss worldwide (Bird and Kaloshian, 2003) and managing these parasites through the manipulation of their microbial symbionts offers an appealing approach. The genus *Pratylenchus* is among the top three most significant plant-parasitic nematodes (Jones et al., 2013), infecting many important crop plants. The conventional methods of controlling this nematode such as crop rotation are generally not successful because of its wide host range. Further, resistant plants are limited to a few crops. Fumigant and non-fumigant nematicides are used to control this nematode, however, there are concerns with these control strategies because of environmental and health-related issues. The discovery of wPpe in this species motivates further exploration of host-endosymbiont interactions in this system centered on its potential as a biological control agent against *P. penetrans*. Furthermore, wPpe is intriguing from a basic biological viewpoint, because of its place as the earliest-branching *Wolbachia* lineage known to date, diverging prior to the *Wolbachia* clades infecting arthropods and filarial nematodes (Brown et al., 2016).

The first description of wPpe was based on a greenhouse *P. penetrans* population, leaving open questions about its prevalence and diversity in field populations, and its potential phenotypic effects on its host. The present study addresses these important gaps in *Wolbachia* symbiosis knowledge using multiple *P. penetrans* field populations in the Northwestern United States. Our specific objectives in this study were to (a) investigate the prevalence of wPpe in *P. penetrans* for a wide range of field populations using a molecular genetic approach, and (b) explore the potential for wPpe to impact host reproduction, which together serve to advance our hypotheses about the possible symbiotic role for this *Wolbachia* strain.

## Materials and Methods

### Nematode Populations and Collection

**Figure 1:**
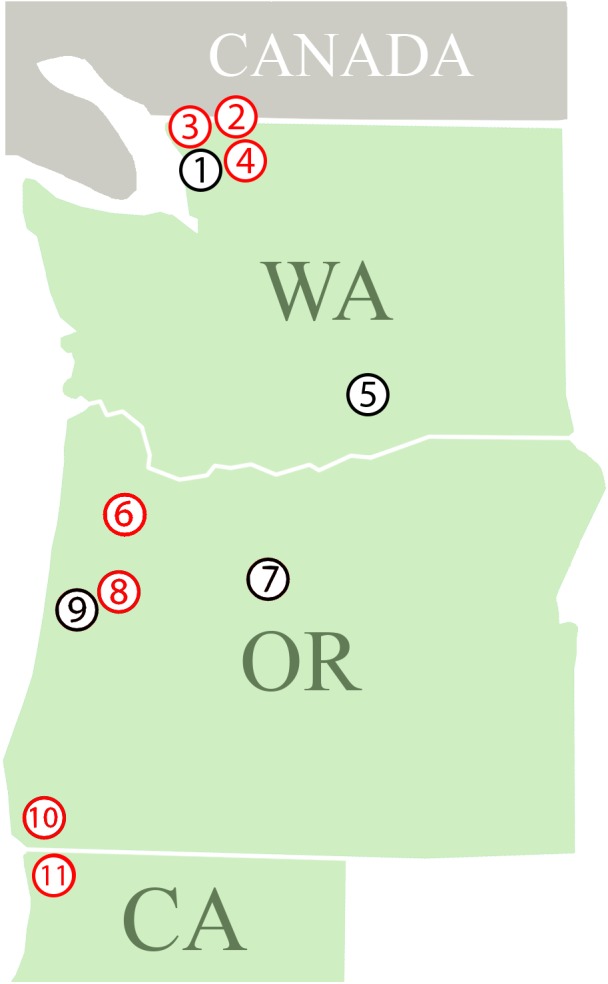
Field locations where *Pratylenchus penetrans* populations were collected. (1–11 = locations; **1:** Lynden; (a–f) – Raspberry, **2:** Sumas – Raspberry, **3:** Custer – Raspberry, **4:** Everson – Raspberry, **5:** Prosser; (a) Cherry, (b) Apple, (c) Mint, **6:** Dayton; (a) Apple, (b) Pear, (c) Strawberry, **7:** Madras – Mint, **8:** Corvallis; (a) Raspberry, (b,c) – Cherry, **9:** Philomath – Mint, **10:** Brookings; (a–e) – Lily, (f–h) – Clover, **11:** Smith River; (a,b) – Lily, (c–e) – Clover. Red: At least one population from this location is infected with *Wolbachia* wPpe, Black: Locations with *Wolbachia* wPpe-uninfected populations.

We collected *P. penetrans* populations from 32 commercial crop fields occurring in 11 different locations in three states (Washington, Oregon, and California) along the West Coast of the United States for this study (Figure 1). The nematode populations were obtained from eight different host plants (Table 1). Additionally, a population of *P. penetrans* maintained in the greenhouse at the USDA-ARS Horticultural Research Crops Laboratory (Corvallis, Oregon) from which wPpe was originally discovered (Brown et al., 2016) was considered. The greenhouse population is maintained on raspberry and was originally collected from raspberry fields in Oregon and Washington. To obtain *P. penetrans* from the fields, multiple root and soil samples were collected from random areas in a field and then combined into a single composite sample. Nematodes were extracted from roots by intermittent mist (Gigot et al., 2013). Root samples were washed free of soil, cut into small pieces (<5 cm) and placed on screens over funnels draining into test tubes and misted at frequent intervals for 5 days. Nematodes were extracted from soil by placing 50 g on a Baermann funnel with nematodes collected after 5 days. *P. penetrans* were collected in water and stored at 4°C until analyzed.

**Table 1:** Occurrence of *Wolbachia* wPpe and *Cardinium* cPpe in *Pratylenchus penetrans* populations.

Location number	Population origins	State	Host plant	*Wolbachia* wPpe	*Cardinium* cPpe
1	Lynden-a	WA	Raspberry	–	–
	Lynden-b	WA	Raspberry	–	–
	Lynden-c	WA	Raspberry	–	–
	Lynden-d	WA	Raspberry	–	–
	Lynden-e	WA	Raspberry	–	–
	Lynden-f	WA	Raspberry	–	–
2	Sumas	WA	Raspberry	+	–
3	Custer	WA	Raspberry	+	–
4	Everson	WA	Raspberry	+	–
5	Prosser-a	WA	Cherry	–	–
	Prosser-b	WA	Apple	–	–
	Prosser-c	WA	Mint	–	+
6	Dayton-a	OR	Apple	–	–
	Dayton-b	OR	Pear	–	–
	Dayton-c	OR	Strawberry	+	–
7	Madras	OR	Mint	–	–
8	Corvallis-a (Greenhouse)	OR	Raspberry	+	+
	Corvallis-b	OR	Cherry	–	–
	Corvallis-c	OR	Cherry	–	–
9	Philomath	OR	Mint	–	–
10	Brookings-a	OR	Lily	+	–
	Brookings-b	OR	Lily	–	–
	Brookings-c	OR	Lily	–	–
	Brookings-d	OR	Lily	–	–
	Brookings-e	OR	Lily	+	–
	Brookings-f	OR	Clover	+	–
	Brookings-g	OR	Clover	–	–
	Brookings-h	OR	Clover	–	–
11	Smith River-a	CA	Lily	–	–
	Smith River-b	CA	Lily	+	–
	Smith River-c	CA	Clover	+	–
	Smith River-d	CA	Clover	–	–
	Smith River-e	CA	Clover	–	–

*“+” indicates endosymbiont presence based on PCR, “-” indicates endosymbiont absence based on PCR. DNA was extracted from ∼200 nematodes for bulk PCR*.

*P. penetrans* field populations collected for this study (*N* = 32) were used to assess the occurrence of endosymbionts (*Wolbachia* wPpe and *Cardinium* cPpe). Depending on the availability, all populations obtained from raspberry (*N* = 10) were used for within-population wPpe prevalence assessment, wPpe 16S ribosomal RNA (rRNA) sequence analysis and *P. penetrans* sex ratio determination.

### Molecular-Genetic Detection of Endosymbionts in *P. penetrans*

Nematodes (*N* = 7,183 total) were screened for the presence of wPpe and cPpe using PCR-based strategies. Two types of DNA samples were screened: (1) DNA prepared from many nematodes originating from the same population (“bulk” samples), (2) DNA prepared from individual nematodes (performed for *P. penetrans* populations obtained from raspberry based on the infection status).

### DNA Extraction

For bulk extractions, *P. penetrans* (*N* = 200) from each population were prepared for DNA isolation in 75 μl of Worm Lysis Buffer (WLB): 1 ml = 880 μl of dH_2_O, 50 μl of 50 mM KCl, 50 μl of 0.05% gelatin, 4.5 μl of 0.45% Tween 20, 10 μl of 10 mM Tris, 3.3 μl of 60 μg/ml Proteinase K and 2.5 μl of 2.5 mM MgCl_2_. Samples were freeze-thawed five times to break the nematode cuticle (frozen at -80°C for >10 min and thawed at room temperature for 5 min) before being digested at 60°C for 90 min, followed by a 95°C incubation for 15 min for DNA extraction. For single-nematode DNA extractions, individual nematodes were picked into 15 μl of WLB, and then followed the same treatment as bulk samples.

### Primer Design, PCR Amplification, and DNA Sequencing

A more sensitive nested PCR which is generally required for *Wolbachia* surveys (Wolfgang et al., 2009) was performed using newly designed wPpe-specific 16S rRNA primers (Supplementary Table S1). Our nested PCR strategy was designed to minimize non-specific primer binding. The first set of primers were designed by aligning *Wolbachia* 16S rRNA sequences from *P. penetrans* and *R. similis* in MEGA6 (Kumar et al., 2008) to amplify a 545 bp fragment. While this primer set matches the 16S rRNA sequence from *P. penetrans* and *R. similis Wolbachia* strains, it also targets other *Wolbachia* strains and a few other proteobacteria. The second primer set specifically targets the 16S rRNA sequence of *Wolbachia* strains from only *P. penetrans* and *R. similis*, and includes several mismatches to sequences from other bacteria, including sister alphaproteobacteria. The second set of primers amplifies a 382 bp fragment of the 16S rRNA gene of *Wolbachia* (primer locations relative to *E. coli* 16S rRNA shown in Supplementary Figure S1). Primers were tested with *P. penetrans* obtained from the greenhouse population where *Wolbachia* was first detected and confirmed by agarose gel electrophoresis and DNA sequencing. *Caenorhabditis elegans*, a nematode known to not harbor *Wolbachia*, was used as a negative control to check for any non-specific binding of the primers.

Nested PCR was started with 1 μl of genomic DNA from bulk DNA extractions or 3 μl of genomic DNA from single nematode DNA extractions. PCR reactions, 50 μl, were performed for *Wolbachia* screens: 5 μl of 10× Taq buffer, 1 μl of 10 μM dNTPs, 2 μl of 10 μM forward primer, 2 μl of 10 μM reverse primer, 0.3 μl of Taq polymerase, 1 or 3 μl of genomic DNA, 38.7 or 36.7 μl of nuclease-free water. The products from the first PCR reaction were diluted 1:10 and used as a template for the second, more specific PCR. To avoid false positives due to contamination, negative controls (sterile water instead of sample DNA) were analyzed for each PCR. All PCR products were visualized on 1.5% agarose gels stained with ethidium bromide.

For wPpe-infected raspberry *P. penetrans* populations (previously identified by bulk DNA PCRs), individual nematode PCR was performed to determine the prevalence of infected nematodes in the source population. Adult females and males were visually identified based on their morphological characters for individual nematode PCR. Individual females (*N* = 100) and males (*N* = 24–100) from each population were analyzed by nested PCR as described above. DNA sequencing of PCR products was performed at the Center for Genome Research and Biocomputing (Oregon State University) to confirm amplification of the correct target.

PCR was performed for bulk DNA extractions using *Cardinium*-specific 16S rRNA primers to screen for cPpe (Brown et al., 2018) followed by DNA sequencing to confirm amplification of the correct target. For the bulk nematode samples shown to be uninfected (by either wPpe or cPpe), three PCR replicates were performed using newly extracted bulk nematode DNA to avoid possible false negatives. For bulk and individual nematode samples, PCR was also performed using *P. penetrans*-specific mitochondrial DNA (mtDNA) primers (Supplementary Table S2), to test for possible false negatives due to inhibitors or improper extraction or digestion of nematode DNA. *P. penetrans* mtDNA primers were designed so that the forward primer targeted *tRNA^Asp^* and the reverse primer targeted *cox1* gene, resulting in an amplicon of ∼840 bp. Samples that did not amplify with mtDNA primers were discarded and not counted in this study. After the gel electrophoresis, the mtDNA PCR products were sequenced and amplification of the correct target was confirmed by blastn searches against NCBI databases.

### wPpe Prevalence Calculation

After the percentages of infected males and females were calculated, total wPpe prevalence in a nematode population was calculated as described below. When the nematode sex ratio is 1:1, the *Wolbachia* percentage was calculated as:

$$Wolbachia\;\%\;=\;\frac{\left(\mathrm{number}\;\mathrm{of}\;\mathrm{males}\;\mathrm{infected}\;+\;\mathrm{number}\;\mathrm{of}\;\mathrm{females}\;\mathrm{infected}\right)}{\;\mathrm{Total}\;\mathrm{number}\;\mathrm{of}\;\mathrm{individuals}}\;\;\;\;\;\;\;\;\;\;\;\;\;\;\;\;\;\;\;\;\;\;\;\;\;\;\;(1)$$

However, when the nematode sex ratio is not 1:1, the male and female nematode percentage in the population were taken into account to estimate the *Wolbachia* percentage. Therefore, the *Wolbachia* percentage was calculated as below.

$$Wolbachia\;\%\;=\;\frac{\left(\%\;\mathrm{males}\;\times \;\%\;\mathrm{infected}\;\mathrm{males})\;+\;(\%\;\mathrm{females}\;\times \;\%\;\mathrm{infected}\;\mathrm{females}\right)}{100}\;\;\;\;\;\;\;\;\;(2)$$

For 1:1 sex ratio, the Equation (2) can be written as below, which is another representation of Equation (1).

$$Wolbachia\;\%\;=\;\frac{\left(\mathrm{50}\;\times \;\%\;\mathrm{infected}\;\mathrm{males})\;+\;(\mathrm{50}\;\times \;\%\;\mathrm{infected}\;\mathrm{females}\right)}{100}\;\;\;\;\;\;\;\;\;\;\;\;\;\;\;\;\;\;\;\;\;\;\;\;\;\;\;\;\;\;\;\;\;\;\;(3)$$

### *Wolbachia* 16S rRNA Sequence Analysis and Phylogenetics

Multiple alignments and phylogenetic analyses of 16S rRNA sequences were performed using MEGA6 (Kumar et al., 2008). DNA sequence multiple alignments were performed using the ClustalW function in MEGA6; the IUB DNA weight matrix was used and the gap-opening and extension penalties were set to 15 and 6.66, respectively (default settings). Reliabilities of resultant multiple alignments were evaluated by visual inspection after running ClustalW.

To evaluate the 16S rRNA diversity of wPpe lineages in different *P. penetrans* populations, we sequenced 54 wPpe 16S rRNA PCR products from individual nematodes obtained from raspberry field populations and the greenhouse population. For the phylogenetic analysis, these were aligned with *Wolbachia* 16S rRNA sequences from four out-groups; plant-parasitic nematode *R. similis* (NCBI accession no: KF059257/EU833482) and filarial nematodes *O. ochengi* (AJ010276), *B. malayi* (AJ010275), and *L. sigmodontis* (FR827944). The outgroups were selected based on results from a previous phylogenetic analysis with 16S rRNA, ftsZ, and groEL genes (Brown et al., 2016). The sequences were trimmed and ∼300 bp of aligned DNA sequences were used for the phylogenetic tree generation. For phylogenetics, model testing was performed in MEGA6 to evaluate the optimal nucleotide substitution model for this data. Then, the optimal model (Kimura 2-parameter model) was utilized to generate a maximum likelihood phylogeny, evaluated with 1000 bootstrap replicates.

### *P. penetrans* Mitochondrial DNA Sequence Analysis

To assess whether wPpe-infected nematode lineages were genetically different from wPpe-uninfected nematodes, we analyzed the segment of the *cox1* mtDNA gene (a fast-evolving marker) from 32 *P. penetrans* field populations and the greenhouse population. DNA extracted from bulk nematode samples were used for this purpose. Nematode mtDNA sequences were aligned, trimmed and then analyzed in MEGA6 as described above.

### Sex Ratio Determination

Ten *P. penetrans* populations obtained from raspberry were used for sex ratio determination. We sampled 300 nematodes per population. For each population, extracted nematodes were placed on a counting slide and observed on an inverted microscope at 40× magnification. Once morphologically identified as *P. penetrans*, adult females were identified by the presence of a vulva, adult males by a bursa/spicule, and juveniles by size and lack of a developed reproductive system. Adult males, adult females, and juveniles were counted under the microscope until 100 individuals per replicate were evaluated; three replicates were counted per population (Supplementary Table S3). Percentage of males and females per replicate were calculated and the average male and female percentages per population were determined. The sex ratio of a population was determined using the average values.

### Statistical Analyses

All statistical analyses were performed using R-Studio version 1.1.423. Chi-square test for homogeneity was performed (Pearson’s Chi-square test), to test for any significant difference in sex ratio between the 10 *P. penetrans* populations obtained from raspberry; Null hypothesis (H_0_): Sex ratio is homogeneous across all *P. penetrans* populations. Chi-square tests for given probabilities (goodness of fit) were performed for each above *P. penetrans* population separately (*N* = 10), to test any deviation from the null expected ratio of 1:1 males and females in the population.

## Results

### wPpe Occurrence and Infection Prevalence in *P. penetrans*

Among the 32 commercial field populations collected, our PCR screen of bulk nematode samples revealed the presence of wPpe in nine *P. penetrans* populations located in Washington, Oregon and California (Table 1). *P. penetrans* populations obtained from raspberry, strawberry, clover, and lily were positive for wPpe while the symbiont was not detected in *P. penetrans* populations collected from mint, cherry, apple, and pear (Table 1). When multiple field samples corresponding to a particular host plant were available, not all of them carried wPpe-infected nematodes indicating that wPpe occurrence is not specific to *P. penetrans* populations infecting specific host plants. wPpe was detected in 40% of the California *P. penetrans* populations, 31% of the Oregon populations, and 25% of the Washington populations. In the four infected raspberry populations, PCR screens of individual nematodes revealed variable wPpe infection levels ranging from 11 to 58% of nematodes analyzed (Table 2). Among infected raspberry *P. penetrans* populations, wPpe was observed in 12–53% of individual females compared to 8–100% of individual males (Table 2).

**Table 2:** Occurrence of *Wolbachia* wPpe and sex ratios of *Pratylenchus penetrans* populations collected from raspberry production fields in Washington, United States.

Population origins	Bulk PCR	Sex ratio	Infected males	Infected females	wPpe
		(mml:F)			prevalence^∗^ (%)
Custer, WA	+	1:1.9	8/100	12/100	10.6
Sumas, WA	+	1:3.7	8/24	44/100	41.6
Everson, WA	+	1:5.9	32/32	51/100	58.2
Greenhouse, Corvallis, OR	+	1:22	9/27	53/100	52.1
Lynden-a, WA	–	1:0.82	NA	NA	NA
Lynden-b, WA	–	1:1.1	NA	NA	NA
Lynden-c, WA	–	1:1.1	NA	NA	NA
Lynden-d, WA	–	1:1.2	NA	NA	NA
Lynden -e, WA	–	1:1.2	NA	NA	NA
Lynden-f, WA	–	1:1.3	NA	NA	NA

^∗^*Derived from equation 2, “+” indicates Wolbachia presence based on PCR, “-” indicates Wolbachia absence based on PCR. DNA was extracted from ∼200 nematodes for bulk PCR, “NA,” not applicable*.

### DNA Sequence Analysis of wPpe 16S rRNA and *P. penetrans* Mitochondrial DNA

**Figure 2:**
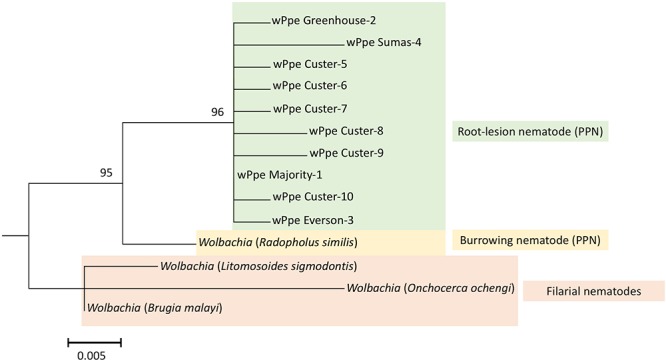
Maximum likelihood phylogeny for *Wolbachia* 16S rRNA; 1000 bootstrap replications. Color indicates *Wolbachia* hosts; Green: *Pratylenchus penetrans*, Yellow: *Radopholus similis*, Red: Filarial nematodes. Corresponding *Wolbachia* hosts are indicated within brackets. *Wolbachia* wPpe numbers represent different 16S rRNA sequence types shown in Supplementary Table S4. wPpe majority represents the 16S rRNA sequence shared by the majority of *P. penetrans*. Scale bar represents the number of substitutions per site. PPN, plant-parasitic nematode.

We sequenced 54 wPpe 16S rRNA PCR products derived from both males and females from all wPpe infected raspberry populations. All amplicons had >99% and 95% sequence identity to the homologous regions sequenced in *Wolbachia* from *P. penetrans* and *R. similis*, respectively (NCBI accession numbers NZ_MJMG01000007 and KF059257, respectively). A *Wolbachia* phylogeny based on the 16S rRNA gene region (Figure 2), using the *R. similis*-infecting *Wolbachia* as an outgroup placed all wPpe sequences into a single clade. Most of the individual nematodes analyzed (45/54) shared an identical wPpe sequence, while there were 9 other unique 16S rRNA sequences (Supplementary Table S4), each occurring in only one nematode. The majority of these alternate sequences contained single nucleotide polymorphisms. While most *P. penetrans* populations contained little genetic variation in wPpe 16S rRNA, one population (Custer) harbored seven different 16S rRNA sequence types (Supplementary Table S4). All 33 *P. penetrans* populations analyzed, including many wPpe-infected and wPpe-uninfected nematodes, shared an identical *cox1* haplotype providing no evidence for an association between wPpe infection status and mitochondrial haplotype.

### Occurrence of *Cardinium* cPpe in *P. penetrans* Populations

cPpe was detected in only 1/32 field populations analyzed (Table 1). The cPpe-infected field population was obtained from a mint field in Washington that did not carry wPpe. The original greenhouse *P. penetrans* population was co-infected with both endosymbionts (wPpe and cPpe). PCR amplicons from cPpe matched the 16S rRNA from *Cardinium* in the plant-parasitic nematode *H. glycines* (NCBI accession number DQ314214) with 92% sequence similarity.

### Sex Ratio Distortion in wPpe-Infected *P. penetrans* Populations

**Figure 3:**
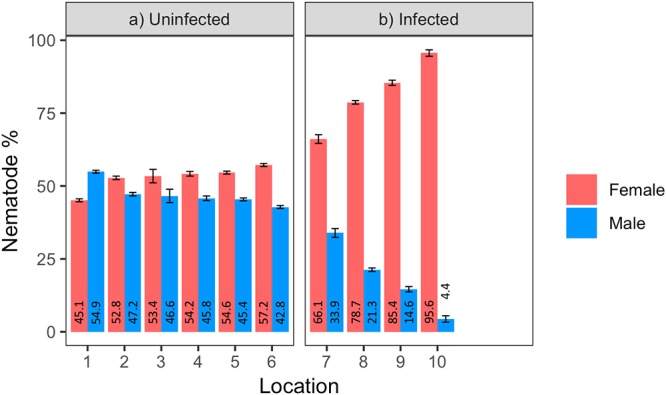
Percentage of males and females in **(a)** uninfected and **(b)** infected *Pratylenchus penetrans* populations (*N* = 100 including juveniles, 3 replicates per population) from raspberry fields in Washington, United States (1–10 = locations; 1–6: Lynden, 7: Custer, 8: Sumas, 9: Everson, 10: Greenhouse). Error bars represent standard error of the mean.

**Figure 4:**
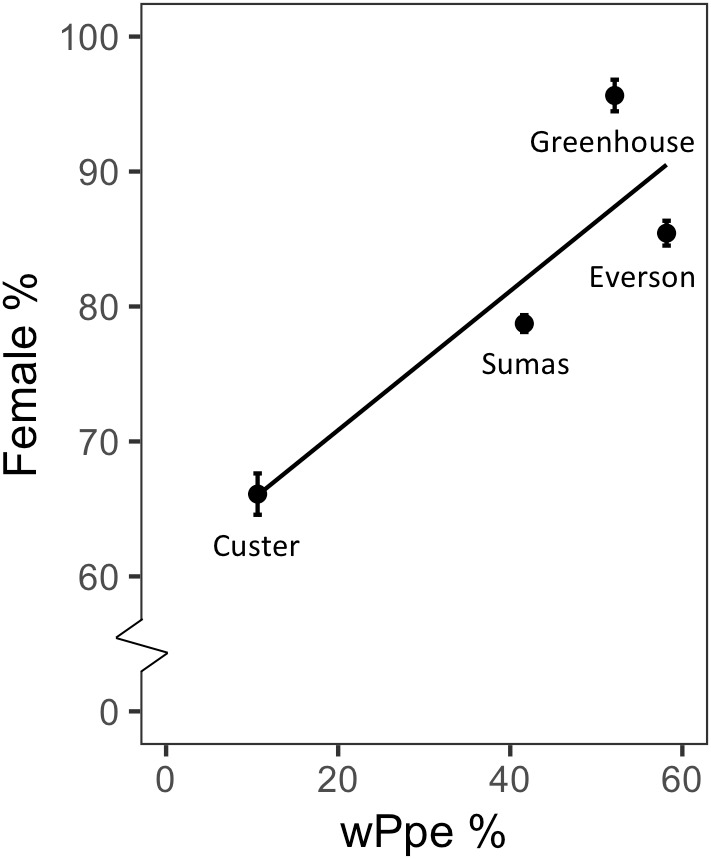
Relationship between sex ratio (percentage females) and percentage of individuals infected with *Wolbachia* wPpe of *Pratylenchus penetrans* populations from raspberry fields in Washington, United States.

Male to female sex ratio in the raspberry-derived *P. penetrans* populations ranged from 1.00:0.82 to 1.00:22.00 (Table 2). Pearson’s chi-square test rejected the null hypothesis that sex ratio is homogeneous across all *P. penetrans* populations obtained from raspberry (*N* = 10) and indicated that among all the *P. penetrans* populations analyzed, there was at least one population with a deviated sex ratio (χ^2^= 120, *df* = 9, *p* < 0.01). The chi-square test for given probabilities (goodness of fit) supported our null hypothesis of male to female sex ratio 1:1 for wPpe-uninfected populations (χ^2^ = 0.6–2.2, *df* = 1, *p* > 0.01, Supplementary Table S5), and rejected the null hypothesis for wPpe-infected populations (χ^2^= 6.7–76.7, *df* = 1, *p* < 0.01, Supplementary Table S5), indicating that there was a sex ratio distortion (either female or male bias) when the nematodes were infected with wPpe. The percentage of female nematodes in the four wPpe-infected populations ranged from 66 to 96%, while the percentage of female nematodes in the six wPpe-uninfected populations ranged from 45 to 57% (Figure 3), indicating sex ratio bias revealed by the chi-square tests was toward females. When the female percentage was graphed against *Wolbachia* percentage, a positive correlative trend was observed; the higher the wPpe prevalence, the higher the degree of female bias in a population (Figure 4).

## Discussion

Studies of natural host-symbiont populations can illuminate critical ecological and evolutionary dynamics relevant in predicting symbiont roles, and help explore the feasibility of symbiont-based biocontrol. Accordingly, this study focused on characterizing natural distribution and impact of the endosymbiont *Wolbachia* wPpe, a newly described, early-diverging lineage in plant-parasitic nematodes (Brown et al., 2016; Denver et al., 2016). The first goal was to characterize the extent of wPpe infection across *P. penetrans* populations collected from commercial crop fields to assess affinity with either reproductive parasite strains or mutualist strains. This question emerged from our previous comparative genomic analyses which could not explicitly predict the functional role of strain wPpe (Brown et al., 2016). In this study, our results clearly demonstrated that some *P. penetrans* field populations do not harbor wPpe: we found only 28% of the populations investigated were infected, a pattern that is more similar to that observed in reproductive parasite *Wolbachia* strains from arthropods (Weinert et al., 2015; Balvín et al., 2018). For *P. penetrans* populations found to harbor wPpe in the bulk screen, our analyses of individual nematodes revealed variable infection levels within populations. However, within-population fixation (i.e., 100% infection levels) was never observed. This observation sharply contrasts with that in well-studied filarial nematodes in which *Wolbachia* is usually found in all individuals of a species (Taylor et al., 2005) due to its obligate mutualistic role. Therefore, these results suggest that wPpe and *P. penetrans* do not engage in an obligate mutualism.

The broadly variable prevalence of wPpe among *P. penetrans* populations could be explained by several causes. Vertical transmission inefficiency poses one possibility. Many *Wolbachia* lineages in other species do not show 100% vertical transmission efficiency from mother to offspring (Kittayapong et al., 2002; Narita et al., 2007), resulting in a proportion of eggs from infected mothers failing to inherit the symbiont. Horizontal transmission offers another potential source for dynamic prevalence data. *P. penetrans* feeds on plant roots, and there is recent evidence that *Wolbachia* has the capacity to transmit horizontally through plants (Li et al., 2017). In *Bemisia* whiteflies, for example, after infected individuals feed on leaves, *Wolbachia* was detected in the plant’s phloem. When *Wolbachia*-free whiteflies subsequently fed on the infected plant leaves, they became infected and were able to vertically transmit endosymbionts to their progeny (Li et al., 2017). Finally, we investigated whether co-infection with a second reproductive manipulator, *Cardinium*, might affect prevalence and sex-ratio bias, but we found only one field population was infected with cPpe, with no *Wolbachia-Cardinium* co-infections, suggesting this secondary symbiont did not contribute to the patterns observed here.

The second goal of this study was to characterize *P. penetrans* sex ratios across field populations to evaluate whether wPpe has the population characteristics of a sex ratio distorter. In most sexually reproducing animals, the sex ratio is maintained at, or close to, 1:1 by frequency-dependent selection (Fisher, 1930). We observed that sex ratios of the wPpe-uninfected *P. penetrans* populations were not significantly different from 1:1 while infected populations were female biased. Furthermore, the female bias in wPpe-infected *P. penetrans* populations was highly significant (*p* < 0.01) and there was a positive correlation between the degree of female bias and the prevalence of *Wolbachia*. This observation was surprising considering past studies have shown that *P. penetrans* is strictly sexual and typically displays a 1:1 sex ratio (Roman and Triantaphyllou, 1969). Sex determination in arthropods and nematodes are often genetically regulated where chromosomal content determines the sex of an individual. However, studies have revealed that in arthropods, sex determination can be manipulated by bacterial endosymbionts, such as *Wolbachia* (Cordaux et al., 2011; Kageyama et al., 2012). For example, *Wolbachia* was associated with extreme female bias in *Cordylochernes* pseudo-scorpions; antibiotic treatment cured females of the *Wolbachia* infection and restored offspring sex ratios to 1:1 (Zeh et al., 2005). In *Oniscus* woodlice and *Eurema* butterflies, females infected with *Wolbachia* produced a higher proportion of females in their broods as compared to uninfected females (Rigaud et al., 1999; Kern et al., 2015).

Our observation of female bias in the wPpe-infected populations could be due to one or more *Wolbachia*-induced reproductive abnormalities such as male killing, feminization of biological males, and parthenogenesis. While there is currently no experimental evidence for male-killing, feminization, or parthenogenesis in wPpe, our data showed a variable prevalence of wPpe in males (8–100%) for the female-biased populations. While infection in males has been reported in arthropods and filarial nematodes (Werren et al., 2008; Slatko et al., 2010), high wPpe prevalence in males would be unexpected under a male-killing phenotype unless the male-killing phenotype occurred late in the development. Similarly, high wPpe prevalence in males would be unexpected in parthenogenesis unless parthenogenesis is weak or incomplete. Incomplete feminization, on the other hand, might be consistent with this data, although males still would not be expected to have higher infection rates than females under this scenario. Our observations might also be influenced by unknown wPpe effects on male nematodes, such as male development rates and dispersion phenotypes. To fully understand the cause of *Wolbachia*-associated sex-ratio bias will require further experimentation using antibiotic treatment and life history comparisons in the *P. penetrans*-wPpe system.

Previously, genomic and tissue distribution analyses of wPpe showed some patterns consistent with this symbiont having the potential to have reproductive manipulation effects (Brown et al., 2016). For example, core and pangenome analyses presented some gene repertoire similarities between wPpe and reproductive manipulators. Further, fluorescent in-situ hybridization experiments revealed a dispersed tissue distribution of wPpe (localized throughout the tissues of *P. penetrans* including the head, pharynx intestine, and ovaries), resembling the tissue distribution patterns found in other *Wolbachia* strains known to be reproductive manipulators (Brown et al., 2016). Conversely, most obligate mutualist *Wolbachia* strains are concentrated in specialized tissues such as the lateral cords (Slatko et al., 2010; Fischer et al., 2011). The genome of wPpe showed no evidence for the presence of CI gene homologs or WO phage genes (Brown et al., 2016), which have recently been implicated as causative of CI (Beckmann and Fallon, 2014; Le Page et al., 2017; Bordenstein et al., 2018). However, wPpe’s evolutionary position as an early-diverging lineage raises the possibility that it might induce CI or similar phenotypes through alternative and unknown genetic mechanisms. In filarial nematodes, *Wolbachia* does not exhibit selfish behaviors such as reproductive manipulation. By contrast, our findings suggest that wPpe might function as a reproductive parasite, similar to *Wolbachia* found in arthropods. Indeed, wPpe has a larger genome than that of filarial nematode *Wolbachia* strains (Brown et al., 2016), supporting the idea that it may harbor more capacity to manipulate hosts.

Another *Wolbachia* strain related to wPpe infects the plant-parasitic nematode *R. similis*, but its symbiotic impacts on the host remain unclear. The report describing *Wolbachia* in *R. similis* suggests an essential function of the endosymbiont based on its prevalence at 100% (Haegeman et al., 2009). However, this was based upon a small sample size, 28 females and 29 males, cultured on carrot discs derived from a population in Uganda. In addition, *Wolbachia* was reported to be present in another *R. similis* population obtained from Indonesia and in a closely related species *R. arabocoffeae* (Haegeman et al., 2009). Additional surveys of *R. similis* field populations might illuminate whether this strain also has variable prevalence similar to that reported here for *P. penetrans.* So far, there is limited information on the presence or absence of *Wolbachia* among plant-parasitic nematode species, with no recent systematic surveys published to date (Brown, 2018). A genome skimming strategy applied to six plant-parasitic nematode species provided no evidence for *Wolbachia* in five of the screened species (Denver et al., 2016) supporting the non-obligatory function of *Wolbachia* in the plant-parasitic nematodes.

## Conclusion

In conclusion, this study demonstrated that *P. penetrans* carrying wPpe occur across a wide geographical range and in a variety of host plants at variable wPpe prevalence that never attained 100%. Therefore, unlike *Wolbachia* symbioses in filarial nematodes, wPpe does not appear to be required and is not an obligate mutualist of *P. penetrans.* The observed correlation between female-biased sex ratio and *Wolbachia* prevalence, suggests that wPpe might act as a reproductive manipulator, but more work is required to confirm this. Future studies should investigate controlled genetic crosses and antibiotic elimination of *Wolbachia*. Such experimental strategies would be, challenging for *P. penetrans*, an obligatory endoparasitic and understudied nematode species. Although it has been difficult to study fitness directly in these obligate migratory endo-parasites of plant roots, it has become possible to culture *P. penetrans* on excised roots, which might open new experimental opportunities for this emerging symbiosis system.

## Author Contributions

SW, DD, AB, and IZ designed the experiments. IZ, DD, DH, and AP collected the root and soil samples. SW, DH, and JK performed the experiments. SW analyzed the data, and wrote the manuscript with input from all the co-authors. All the authors approved the final version of the manuscript.

## Conflict of Interest Statement

The authors declare that the research was conducted in the absence of any commercial or financial relationships that could be construed as a potential conflict of interest.
