# Supplementary material for: Variable Abundance and Distribution of Wolbachia and Cardinium Endosymbionts in Plant-Parasitic Nematode Field Populations
Source: Front Microbiol. 2019 May 7;10:964. doi: 10.3389/fmicb.2019.00964 (PMC6513877; doi:10.3389/fmicb.2019.00964)
Supplement: Supplementary file 1 [file Data_Sheet_1.PDF]

Supplementary Material

TABLE S1 PCR primers and conditions used for prevalence survey and sequencing of *Wolbachia* wPpe in *Pratylenchus penetrans*.

	Primer Name	Sequence	Thermal Cycle Conditions
1st PCR	Wol_16S_F	5’ GGT AGG GTA ATG GCT TAC CAA GG 3’	95°C – 2 min<br>95°C – 30 sec<br>52°C – 20 sec } X 36<br>72°C – 2 min<br>72°C – 10 min
	Wol_16S_R	5’ TTC ATC GTT TAC AGC GTG GA 3’	
2nd PCR	Wol_281B_F	5’ AAT GGC TTA CCA AGG YWA TGA TCT 3’	95°C – 3 min<br>95°C – 30 sec<br>58°C – 20 sec } X 36<br>72°C – 1 min<br>72°C – 10 min
	Wol_692_R	5’ TCC TCT TTC AAT CTC TAG ATT AG 3’	

*This PCR was performed in two steps; the products from the first PCR reaction were diluted 1:10 and used as a template for the second, more specific PCR.

TABLE S2 PCR primers and conditions for survey and sequencing of *Pratylenchus penetrans* mitochondrial DNA.

Primer Name	Sequence	Thermal Cycle Conditions
Pp_mt_tRNA_F	5’ TCTAAGTACTACCTGAC 3’	95°C – 2 min<br>95°C – 30 sec<br>52°C – 20 sec } X 36<br>72°C – 2 min<br>72°C – 10 min
Pp_mt_coxI_R	5’CCAATTCTCTTTATAGCGCAAC 3’	

TABLE S3 Number of male, female, juvenile nematodes and *Wolbachia* prevalence from field and greenhouse populations of *Pratylenchus penetrans* obtained from raspberry.

**Population origin**	**Trial**	**Number of juveniles**	**Number of adult males**	**Number of adult females**	***Wolbachia* prevalence (%)**	
Custer, WA	1	79	7	14	10.7	Infected *P. penetrans* Populations
	2	81	6	13	10.5	
	3	81	7	12	10.5	
Everson, WA	1	61	5	34	57.3	
	2	56	7	37	58.8	
	3	60	6	34	58.4	
Sumas, WA	1	60	9	31	41.5	
	2	52	10	38	41.7	
	3	61	8	31	41.7	
Greenhouse, Corvallis, OR	1	67	1	32	52.4	
	2	70	2	28	51.7	
	3	71	1	28	52.3	
Lynden-a, WA	1	73	15	12	0%	Uninfected *P. penetrans* Populations
	2	71	16	13		
	3	74	14	12		
Lynden-b, WA	1	29	33	38		
	2	34	32	34		
	3	40	28	32		
Lynden-c, WA	1	50	22	28		
	2	44	25	31		
	3	53	24	23		
Lynden-d, WA	1	47	24	29		
	2	43	27	30		
	3	44	25	31		
Lynden-e, WA	1	44	26	30		
	2	44	25	31		
	3	49	23	28		
Lynden-f, WA	1	62	16	22		
	2	68	14	18		
	3	67	14	19		

TABLE S4 *Wolbachia* wPpe16S rRNA sequence variation from field and greenhouse populations of *Pratylenchus penetrans* obtained from raspberry. The original wPpe 16S sequence is 1505 bp long, as annotated in NCBI accession no: NZ_MJMG01000007.

16S Sequence type	Number of individuals	Number of SNPs	Positions of the SNPs	Populations of origin
1	45	0	N/A	Custer, Everson, Sumas, greenhouse
2	1	1	1111	greenhouse
3	1	1	1174	Everson
4	1	3	1014, 1017, 1155	Sumas
5	1	1	982	Custer
6	1	1	952	Custer
7	1	1	1048	Custer
8	1	2	977, 1146	Custer
9	1	2	1003, 1182	Custer
10	1	1	945	Custer

TABLE S5 Results of chi-square test for given probabilities (chi-square test for goodness of fit).

Population origins	Bulk PCR	χ^2	p-value
Custer, WA	+	6.6667	9.823e-3
Sumas, WA	+	41.961	9.313e-11
Everson, WA	+	61.537	4.346e-15
Greenhouse	+	76.696	2.2e-16
Lynden-a, WA	_	0.7805	0.3770
Lynden-b, WA	_	0.6142	0.4332
Lynden-c, WA	_	0.7908	0.3738
Lynden-d, WA	_	1.1807	0.2772
Lynden-e, WA	_	1.3804	0.2400
Lynden-f, WA	_	2.1845	0.1394

df = 1, probability of males = 0.5, probability of females = 0.5, "+" indicates *Wolbachia* presence, "-" indicates *Wolbachia* absence.

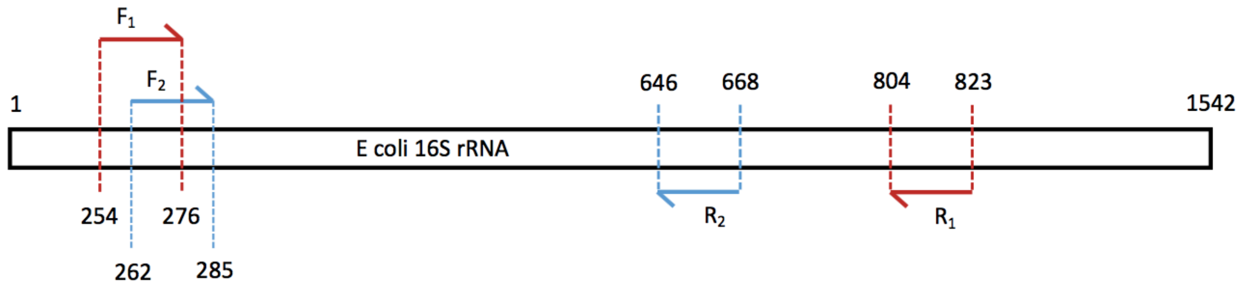

FIG S1 Schematic of *Wolbachia* wPpe 16S rRNA primer positions relative to *E. coli* 16S rRNA (GenBank accession no: BA000007). F1: Wol_16S_F1, R1: Wol_16S_R1, F2: Wol_281B_F, R2: Wol_692_R.
